# Planning abilities of wild chimpanzees (*Pan troglodytes troglodytes*) in tool-using contexts

**DOI:** 10.1007/s10329-023-01106-4

**Published:** 2023-12-16

**Authors:** Stephanie Musgrave, David Koni, David Morgan, Crickette Sanz

**Affiliations:** 1 Department of Anthropology, University of Miami, P.O. Box 248106, Coral Gables, FL 33124-2005 USA; 2 Wildlife Conservation Society, Congo Program, B.P. 14537 Brazzaville, Republic of Congo; 3 Fisher Center for the Study and Conservation of Apes, Lincoln Park Zoo, 2001 N. Clark Street, Chicago, IL 60614 USA; 4 Department of Anthropology, Washington University in Saint Louis, 1 Brookings Drive, Saint Louis, MO 63130 USA

**Keywords:** Cognition, Tool use, Tool manufacture, Tool set, Termite fish

## Abstract

Planning is a type of problem solving in which a course of future action is devised via mental computation. Potential advantages of planning for tool use include reduced effort to gather tools, closer alignment to an efficient tool design, and increased foraging efficiency. Chimpanzees (*Pan troglodytes troglodytes*) in the Goualougo Triangle use a variety of different types of tools. We hypothesized that procurement strategy (brought to the termite nest, manufactured or acquired at the termite nest, or borrowed from others) reflects planning for current needs, with tool transport behavior varying by tool type and by age and sex class. It is also possible that chimpanzees anticipate the need for tools at future times, which would be evidenced by transporting multiple tool types for a sequential task. One year of video recordings at termite nests were systematically screened for tool procurement; data comprised 299 tool procurement events across 66 chimpanzees. In addition, we screened video recordings of leaf sponging and honey gathering, which resulted in another 38 procurement events. Fishing probes, which are typically used during a single visit, were typically transported to termite nests, while puncturing tools, which are durable and remain on site, were more often acquired at termite nests. Most tools transported in multiples were fishing probes, perhaps in anticipation that a single probe might not last through an entire foraging bout or might be transferred to another chimpanzee. We further documented that chimpanzees transported tool sets, comprising multiple different tool types used in sequence. Mature chimpanzees transported tools more often than did immatures. These observations suggest that chimpanzees plan tool use flexibly, reflecting the availability of raw materials and the likelihood that specific tool types will be needed for particular tasks. Developmental studies and further integration of behavioral, spatial, and archaeological data will help to illuminate the decision making and time depth of planning associated with tool technologies in living primates and hominin ancestors.

**Supplementary Information:**

The online version contains supplementary material available at 10.1007/s10329-023-01106-4.

## Introduction

The ability to plan for the future confers numerous adaptive advantages, and it is a fundamental part of human life (Suddendorf and Corballis 1997). An evolutionary perspective on planning requires identification of the associated cognitive underpinnings as well as comparative assessments of future-oriented behavior across the animal kingdom (Osvath and Martin-Ordas 2014). While prospective behavior has been documented across a wide range of taxa, particularly corvids and primates (Raby and Clayton 2009), debate persists about the extent, flexibility, and mechanisms of these planning abilities in nonhumans (Redshaw and Bulley 2018).

The cognitive underpinnings of planning likely vary relative to how far in advance planning occurs, as well as the complexity of the future behavior that an individual must mentally represent, among other factors. From one perspective, planning ability emerges exclusively from the episodic cognitive system (Tulving 1972). Episodic memory involves mentally revisiting specific past experiences, which enables projection of oneself into, and thus appropriate handling of, future scenarios (Krause and Sanz 2019). Behavioral and electrophysiological measures suggest a wide range of species may be able to act on the basis of episodic or “episodic-like” memory (Crystal 2018). According to the Bischof–Köehler (1985) hypothesis, true planning also involves “episodic foresight,” the ability to project oneself into a future state where one’s needs or desires differ with those currently present. Suddendorf and Corballis (1997) hypothesize that this package of abilities, termed “mental time travel,” is unique to humans.

The primacy of the episodic cognitive system in governing future-oriented behavior is debated, however, and this system may not impact future planning in the same way across individuals. For example, experiments requiring participants to disengage from a current state (e.g., satiation on a particular food) in order to plan for the future suggest that episodic memory abilities in humans may in some cases vary negatively with the ability to project to a future state of need. Individuals who were most successful remembering small details of past events showed greater difficulty disengaging from their current state when planning for the future (Cheke and Clayton 2019). Myriad other cognitive components—e.g., semantic and working memory, inhibitory control—are likely also involved in future-oriented behavior (Osvath and Martin-Ordas 2014; Suddendorf and Corballis 2007). In addition, the ability to imagine feeling differently than one does at present is not necessarily synonymous with the ability to mentally simulate a future action (Byrne et al. 2013). Increasing the breadth and depth of research on future-oriented behavior in nonhumans is essential for illuminating the diversity of related cognitive abilities and the evolutionary basis of such skills (Krause and Sanz 2019), as well as the environmental interactions that support the development and expression of cognitive abilities, including planning (Boesch 2022).

Tool behavior can potentially render aspects of animals’ thought processes more “visible” by increasing the salience of relevant abilities, including planning. For example, the preparation of raw materials to achieve particular end goals can serve as a visible manifestation of anticipatory thought (Byrne et al. 2013). Further, examining tool behavior in living animals is invaluable for inferring the role of planning and other cognitive capacities in the technologies of human ancestors. Evidence for planning abilities has been documented in captive and experimental conditions, where great apes both save (Dufour and Sterck 2008; Mulcahy and Call 2006; Osvath and Karvonen 2012; Osvath and Osvath 2008) and produce tools for future use, including manufacture of multiple functional tools (Bräuer and Call 2015). Critics of a planning interpretation suggest that these actions could result from merely learned associations between objects and past rewards rather than true planning, and they further highlight ways in which apes do not seem sensitive to the specific future conditions they will encounter (Redshaw and Bulley 2018). For example, apes in the Bräuer and Call (2015) experiment produced fewer (on average fewer than two) than the optimal number of tools (at least eight) that would be needed for a future task. Thus, there is still ample work required to elucidate flexible planning capacities in great apes and to replicate positive findings (Redshaw et al. 2018).

Cognitive abilities develop in association with environmental experience, and so to understand planning, it is essential to examine relevant behaviors across populations living in different ecological conditions (Rosati et al. 2014, Boesch 2020). In wild populations, individuals must carefully manage energetic investment in foraging while considering a multitude of variables. In the context of foraging tool use, planning could confer adaptive benefits if it helps maximize energetic gains, for example, by reducing effort required to procure tools and facilitating efficient alignment to sophisticated tool forms (Sanz et al. 2009). As an indicator of planning, tool transport is illuminating because it suggests that animals potentially anticipate a forthcoming task and the appropriate requirements for executing it (Byrne et al. 2013).

Among wild nonhuman primates, chimpanzees (Boesch and Boesch 1984a; Goodall 1964; McGrew 1974; Nishida 1973; Sanz et al. 2004) and capuchins (Visalberghi et al. 2015) have been documented selecting and/or manufacturing tools in advance and transporting them to a tool site. At Taï, chimpanzees take multiple variables into account when selecting which hammers to use for nut cracking (Sirianni et al. 2015). The chimpanzees prefer heavier hammers when they are closer to anvils (which are at fixed locations), but they switch to a “prefer lighter” criterion as the distance to the anvil increases (Boesch 2022). Given that they exhibit this flexible, conditional selection of tools even when the anvil to be used is out of view (Boesch and Boesch 1984a), they may use memories of nut-cracking sites when preparing for tool use (Sirianni et al. 2015).

Chimpanzees also transport perishable tools. In the context of ant fishing at Mahale, for example, chimpanzees have been documented manufacturing tools in advance and then carrying these to an ant-infested tree (Nishida 1973). Typically, transport distances are short, as tools are manufactured from plants within close proximity to anting locations, often from branches of the tree in which the nest is located. However, on some occasions, chimpanzees transported tools over 10 m, with one transport event of approximately 70 m (Nishida 1973). Several species of grasses and vines with particularly useful dimensions and material properties were always transported in advance to nests (Nishida and Hiraiwa 1982). In termite gathering, chimpanzees may also gather raw material sources that are out of view of a termite nest (Almeida-Warren et al. 2017; Goodall 1964; Pascual-Garrido 2018). In the Issa Valley, Tanzania, for example, chimpanzees gathered plants up to 33 m from termite mounds, and over half of plant sources were at a distance of 10 m or further from the nest, often out of view of the nest (Almeida-Warren et al. 2017). However, it is not necessarily clear whether chimpanzees encounter a termite nest and then depart to gather materials, or whether they arrive with tools already in their possession.

**Fig. 1:**
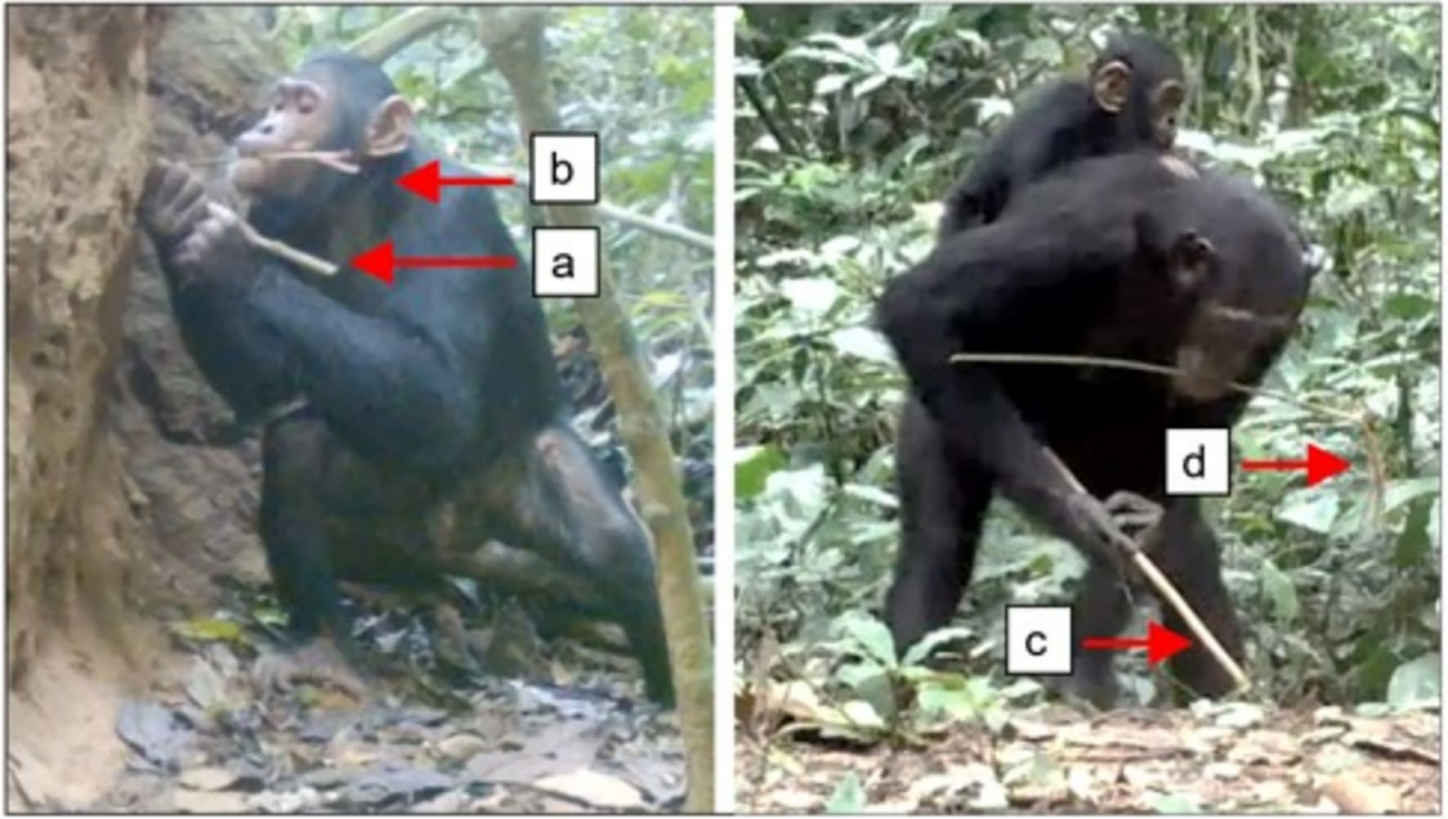
Tool sets to gather termites at epigeal (left) and subterranean (right) nests. A subadult male clears a termite exit hole with a perforating twig **a** before using a fishing probe **b** to extract termites. An adult female tunnels into the earth with a woody puncturing stick **c**; the brush-tip fibers are visible on the end of the fishing probe **d** she holds in her mouth

Central chimpanzees (*Pan troglodytes troglodytes*) employ multiple, perishable tool sets—which involve two different tool types used sequentially to achieve a goal (Brewer and McGrew 1990)—to gather invertebrate resources such as termites, honey, and ants (Bermejo and Illera 1999; Boesch et al. 2009; Estienne et al. 2017; Fay and Carroll 1994; Sanz et al. 2004, 2010; Sanz and Morgan 2007, 2009). In the Goualougo Triangle, Republic of Congo, chimpanzees have been observed manufacturing and using two distinct tool sets to gather termites of the genus *Macrotermes* from epigeal (above-ground) and subterranean nests. At epigeal nests, chimpanzees use their fingers or a perforating twig to open termite exit holes on the nest surface before inserting an herbaceous probe into the nest to termite fish. In the subterranean setting, chimpanzees use a woody puncturing stick to create a tunnel into underground nest chambers before using a fishing probe to extract termites (Fig. 1). In both contexts, the chimpanzees fray the ends of the smooth, pliable fishing probes to a brush tip. Rather than resulting from a byproduct of use, the brush tip is an intentional modification that improves the efficiency of the tool at gathering insects (Sanz et al. 2009). Durability is a key difference between these tool types: herbaceous fishing probes are more fragile and typically used during a single visit, while the woody puncturing sticks can withstand many uses and last for weeks or months.

Goualougo chimpanzees are highly selective for plant species chosen to manufacture some tool types, including fishing probes and puncturing sticks. They do not show such selectivity in selecting leaves to sponge water or branches to manufacture beehive pounding clubs (Sanz and Morgan 2007). Ninety-eight percent of puncturing sticks are typically manufactured from *Thomandersia hensii*, which has straight, rigid, and durable branches. While prevalent, *T. hensii* is not always available immediately near the termite nest, and chimpanzees do gather materials from this species tens of meters away and out of visibility of the nest (Sanz et al. 2004; Sanz and Morgan 2007). More than 96% of fishing probes are manufactured from just one or two species of herb within the Marantaceae family. While Marantaceae is the dominant undergrowth, the particular species preferred by chimpanzees are not the most abundant (Sanz and Morgan 2007). Within a 20 m radius of termite nests, the closest preferred fishing probe material is on average at a distance of 6.35 ± 5.33 m (Sanz and Morgan 2013). The raw material requirements and the obligate use of tool sets for termite gathering in comparison to the flexibility in materials used for leaf sponging and honey gathering in this population make it an ideal context in which to examine planning in great apes, as tool users may have to mentally represent multiple, temporally separated future steps, each involving use of tools that adhere to a different template (Byrne et al. 2013; Martin-Ordas 2020). Previous research described 45 cases of chimpanzees arriving at termite mounds with fishing probes and/or puncturing sticks, with chimpanzees more often transporting fishing probes (Sanz et al. 2004). Procurement of other tool types has not been reported. In this study, we examined more broadly what strategies chimpanzees use to procure tools for termite gathering, leaf sponging, and honey gathering for individuals across the lifespan, to better assess the scope and development of their flexible planning abilities.

We hypothesized that chimpanzees planned for current needs (i.e., hunger or desire for termites). Thus, we anticipated differences in the transport and use of the different tool types with respect to whether tools were brought to the tool site (termite nest, beehive, water basin), manufactured or acquired at the site after arrival, or transferred from others. We also expected that chimpanzees would anticipate the need for tools at future times, which would be evidenced by transporting multiple tool types for a sequential task. Specifically in the termite-gathering context, we predicted that there would be significant differences in how often the more fragile fishing probes would be transported to nests and that they would be more often transported in multiples compared to other tool types. The more durable puncturing sticks, in contrast, are conserved at nests between visits, and so chimpanzees were expected to transport them less often and rarely in multiples. Given that both a puncturing stick and a fishing probe must be used to gain access to termites at subterranean nests, we anticipated that chimpanzees would at least sometimes transport this tool set, demonstrating preparation for both the first and subsequent stages of this task. Unlike fishing probes and puncturing sticks, perforating twigs are not manufactured from specific raw materials and are not required at epigeal nests, and so transport of this tool type was not anticipated. For beehive pounding, we predicted that mature individuals would more frequently transport tools than immature chimpanzees. Other types of tools used for honey gathering are not obligate, and so, like perforating twigs, may be gathered at the site as needed. We also did not expect leaf sponging tools to be transported, as chimpanzees typically use any leaves available at the water basin.

Past studies on a range of tool-using taxa have documented ontogenetic changes as well as sex differences in tool behavior (for example with respect to skill or frequency of tool use), but the influence of these variables on tool procurement strategies has not previously been investigated in this context. Particularly for complex tool tasks, mastery of tool use and manufacture can take many years. In the Goualougo Triangle, for example, all chimpanzees termite fish successfully by 2.9 years, but they do not manufacture brush-tipped fishing probes until later infancy or juvenility, at an average age of 4.3 years. Development of the use of tool sets extends even further, into adolescence (Musgrave, Lonsdorf, Morgan, and Sanz, 2020). We thus predicted that mature compared with immature chimpanzees would be more likely to plan their tool use, evidenced by them more frequently arriving with tools to the sites where the tools will be used. Sex differences in frequency of adult tool use and/or use of particular tool types are reported for chimpanzees (e.g., Boesch and Boesch 1984b; Goodall 1986; Pruetz and Bertolani 2007), captive bonobos (Boose et al. 2013; Gruber et al. 2010), macaques (Gumert et al. 2011), and capuchins (e.g., Falótico and Ottoni 2014; Spagnoletti et al. 2011). It is unclear, however, whether such differences extend to the cognitive underpinnings of tool behaviors and if so, at what age such differences appear. Thus, we assessed whether females and males differed in procurement strategy. We also specifically examined whether adult females with offspring more often transported multiple tools, given past observations that adult females sometimes transfer tools to their offspring over the course of termite-gathering sessions (Musgrave et al. 2016; Musgrave et al. 2020a, b).

## Materials and methods

### Study site and subjects

Chimpanzee observations were conducted in the Goualougo Triangle, located in the Nouabalé-Ndoki National Park (N 2°05−3°03; E 16°51−16°56) in the Republic of Congo.

The study site encompasses 380 km^2^ of evergreen and semi-deciduous lowland forest; the altitude ranges from 330 m to 600 m. There is a primary rainy season from August to November and a short rainy season in May.

Chimpanzees were identified based on unique individual characteristics, and age/sex class of chimpanzees was scored based on known birthdates as well as physiological and developmental criteria (Boesch and Boesch-Achermann 2000; Estienne et al. 2019; Goodall 1968, 1986; Plooij 1984). Tool procurement by known individuals was observed across age/sex classes for termite gathering, honey gathering, and leaf sponging (Table 1).

**Table 1:** Number of unique individuals observed by age/sex class and tool context

Age class	Sex	Tool context		
		Termite gathering	Honey gathering	Leaf sponging
Adult*	Male	21	1	
	Female	21	2	3
Subadult	Male	2		2
	Female	5	1	2
Juvenile	Male	4	2	1
	Female	11	2	
	Unknown		1	
Infant	Male	1		
	Female	1		
Total per category		66	9	8

*Adult male: 15+ years old; adult female: 15+ years old or first offspring; subadult: 10–15 years old; juvenile: 5–10 years; infant: 0–5 years

### Data collection and reliability

Remote video recording devices have been employed for surveillance of chimpanzee presence at termite nests in the Goualougo Triangle since 2003 (Sanz et al. 2004). A complete year of chimpanzee termite-gathering behavior at subterranean, epigeal, and subterranean-epigeal nests was screened, comprising approximately 25 h of video footage recorded between April 2005 and May 2006. Thirty-seven termite nests were also surveyed to determine the location of the closest available raw materials for fishing probe manufacture. To broaden the comparison of tool types used by this population, we screened video recordings from daily follows of chimpanzees for tool use in honey gathering and leaf sponging. All video was screened and scored with INTERACT software (Mangold 2021).

Every occurrence of a chimpanzee possessing a tool was recorded. For each unique tool possessed by a chimpanzee, scored variables included termite nest type, tool type, how the chimpanzee came to possess the tool (the tool “origin”, Table 2 and Additional file 1: Video S1), as well as whether the tool was transported in multiples and/or as part of a tool set. Cohen’s κ was calculated between two independent observers to ensure adequate interobserver reliability for key variables (Landis and Koch 1977) before proceeding with coding. Interobserver reliability for termite nest type was κ = 1.00; tool type (fishing probe, perforating twig, puncturing stick, no tool, not visible) κ = 0.87; and for tool origin (arrived, acquired, gathered and manufactured, transferred, unknown origin, no tool present, or origin not visible) κ = 0.71. Observers consulted on the final dataset to ensure consensus in how observations were assigned (sensu Humle and Matsuzawa 2002).

**Table 2:** Tool origin category definitions

Tool origin category	Definition
Arrived	Chimpanzee brings tool to site where it will be used (termite nest, beehive, water basin). Evidence of arrival comprises an individual locomoting toward site at the start of a tool using episode while transporting a tool or tool raw material. Other supporting criteria include: quadrupedal posture, visual scanning of the area, and/or approaching the camera. Immature individuals may dismount from an adult female’s back while transporting a tool or tool raw material
Gathered and manufactured	After arriving at the tool use site, the chimpanzee gathers raw material and manufactures a tool. The chimpanzee may gather raw material from the immediate vicinity of the nest or travel to surrounding vegetation and return with raw material to manufacture a tool
Acquired	After arriving at the tool use site, the chimpanzee picks up previously detached raw material or a previously manufactured tool
Transferred	After arriving at the tool use site, the chimpanzee acquires a tool from a second individual. The chimpanzee may request and be given a tool, be passively permitted to take a tool, or steal a tool from another individual^a^
Unknown origin	The origin of the tool cannot be determined, for example, because the chimpanzee first becomes visible after repositioning from another location at the nest, at which point the individual already has a tool
No tool	The individual does not possess a tool at any time while present at the tool use site
Not visible	Tool and/or individual present in a clip is not adequately observable to score tool origin

^a^See Musgrave et al. 2016, and Musgrave et al. 2020a, b, for further details

To investigate whether procurement strategy varied according to tool type for termite harvesting, Fisher’s exact test was used to assess the association between the variables tool type and tool origin category. The sample sizes associated with the other tool types precluded their inclusion in this analysis, and so these findings are reported using descriptive statistics. We also report descriptive statistics regarding transport of multiple tools and tool sets. Chi-square tests were conducted to compare procurement strategies between age classes and between the sexes for fishing probes, the most numerous tool type represented. For these analyses, we assessed whether chimpanzees arrived with these tools versus procured them via any other means (gathered and manufactured after arrival, acquired after arrival, or via transfer). For analysis of age, the comparison is between the categories of mature (adult and subadult) and immature (infant and juvenile) individuals, and for analysis of sex, exclusively mature females and mature males are compared. Two observations of fishing probe procurement were excluded from age analyses and from Table 3 because age class could not be definitively assigned. We report descriptive statistics for assessments of age and sex across the other tool types. The significance threshold was set at 0.05, and all analyses were conducted in R (version 4.2.2) (R Core Team, 2021).

**Table 3:** Number and proportion of events per tool origin category and age class, for each tool type

	Tool origin				
	Arrived	Gathered and manufactured	Acquired	Transferred	Total no. of tools
Fishing probe					
Adult	120 (83.9%)	12 (8.4%)	9 (6.3%)	2 (1.4%)	143
Subadult	40 (88.9%)	3 (6.7%)	1 (2.2%)	1 (2.2%)	45
Juvenile	28 (60.9%)	4 (8.7%)	6 (13.0%)	8 (17.4%)	46
Infant	0	2 (66.7%)	1 (33.3%)	0	3
Puncturing stick					
Adult	4 (19.0%)	2 (9.5%)	13 (61.9%)	2 (9.5%)	21
Subadult	4 (23.5%)	0	10 (58.5%)	3 (17.6%)	17
Juvenile	0	1 (33.3%)	2 (66.7%)	0	3
Infant	0	0	1 (100%)	0	1
Perforating twig					
Adult	0	7 (43.8%)	9 (56.3%)	0	16
Subadult	0	0	0	0	0
Juvenile	0	1 (100%)	0	0	1
Infant	0	1 (100%)	0	0	1
Beehive pounding club					
Adult	4 (66.7%)	2 (33.3%)	0	0	6
Subadult	1 (100%)	0	0	0	1
Juvenile	1 (14.3%)	5 (71.4%)	0	1 (14.3%)	7
Infant	0	0	0	0	0
Other honey gathering tool					
Adult	0	2 (100%)	0	0	2
Subadult	0	0	0	0	0
Juvenile	0	7 (87.5%)	1 (12.5%)	0	8
Infant	0	0	0	0	0
Leaf sponge					
Adult	0	3 (100%)	0	0	3
Subadult	0	9 (100%)	0	0	10
Juvenile	0	1 (100%)	0	0	0
Infant	0	0	0	1 (100%)	1
Total per category	202	62	53	18	335

### Ethical note

This research project was approved by the Institutional Animal Care and Use Committee of Washington University in St. Louis. All field protocols were also conducted in accordance with the legal requirements of the Republic of Congo where the research was conducted. This study was also endorsed by the Nouabalé-Ndoki Foundation and the Wildlife Conservation Society’s Congo Program. Finally, the authors have no conflict of interest.

## Results

### Variation in procurement strategy according to tool type

Tool origin was definitively assigned for *n* = 337 tool events, including *n* = 239 fishing probes, *n* = 42 puncturing sticks, *n* = 18 perforating twigs, *n* = 14 beehive pounding clubs, *n* = 10 other types of honey gathering tools, and *n* = 14 leaf sponges. When considering all tool types together, arrivals comprised the largest proportion of events (60.5%), followed by gathering and manufacturing on site (18.4%), acquisition after arrival (15.7%), and transfer (5.3%). For termite-gathering tools specifically, the largest proportion of procurement events was composed of arrivals (66.2%), followed by acquisition after arrival (17.4%), gathering and manufacturing on site (11.0%), and transfer (5.4%). Transport of fishing probes was documented for 190 events, constituting the vast majority (96%) of the arrival events.

**Fig. 2:**
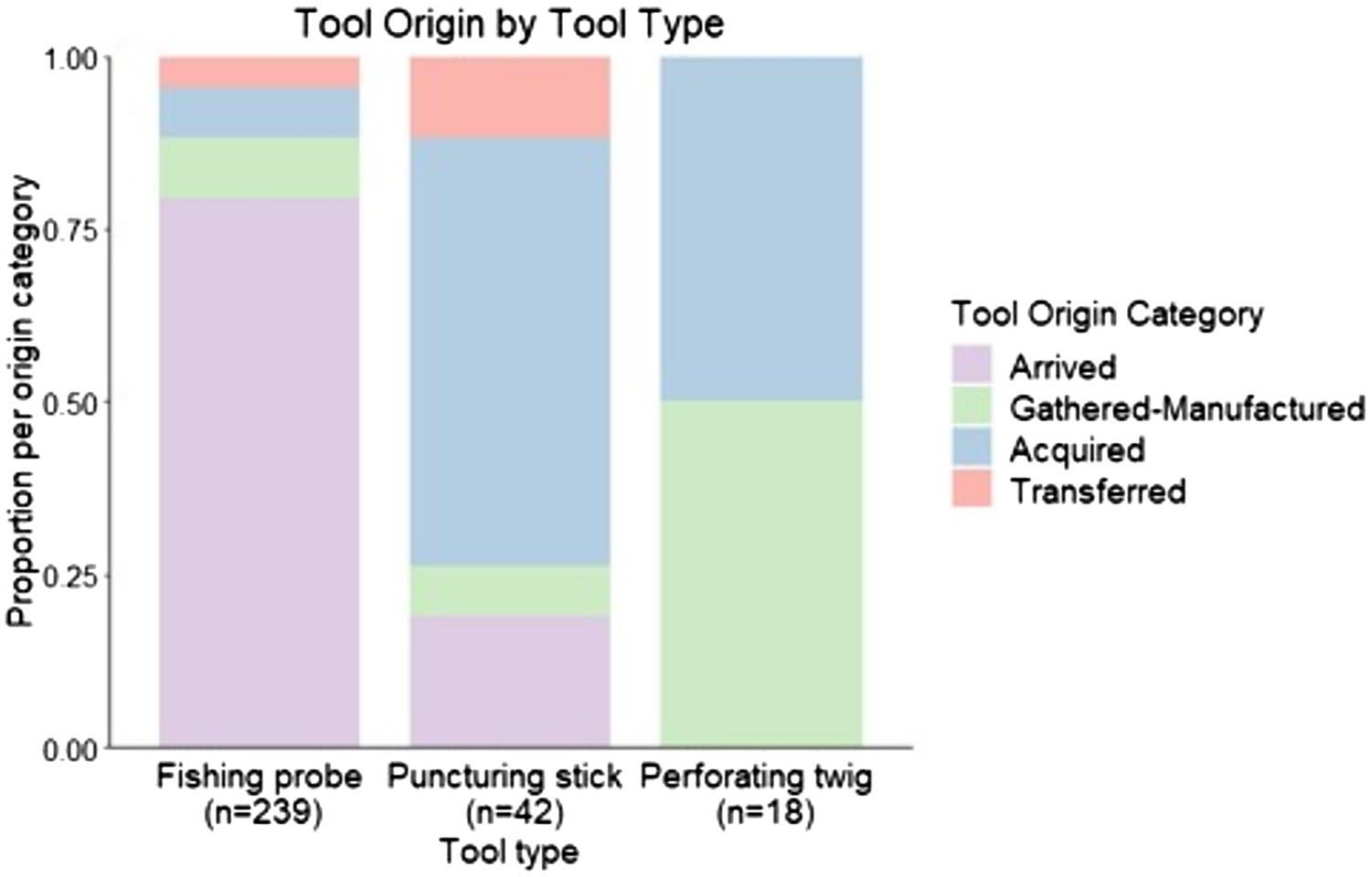
Tool origin varied according to tool type. Chimpanzees usually transported fishing probes to nests, while puncturing sticks were most often acquired after arrival. Perforating twigs were never transported in advance; instead, chimpanzees picked up or made these tools once at the nest

There was a significant association between termite-gathering tool type and tool origin category (Fisher’s exact test, *p* < 2.2 × 10^−16^). Chimpanzees more often arrived at termite nests with fishing probes as opposed to procuring these tools via another method (gathering and manufacture on site, acquiring, or transfer). In contrast, chimpanzees were more likely to acquire puncturing sticks after arrival at the nest, rather than procuring these tools via another strategy. Chimpanzees did not arrive at epigeal nests with puncturing sticks, only arriving with this tool type at subterranean nests. Chimpanzees never arrived transporting perforating twigs; after arrival at the nest, they typically manufactured these or picked up a suitable nearby twig when necessary (Fig. 2). On three occasions, chimpanzees perforated a termite exit hole by reversing the orientation of their fishing probe to use the unmodified end of the tool to clear the tunnel; the fishing probe thus served as a multifunction tool.

### Transport of multiple tools and tool sets

Of the 190 fishing probes transported to termite nests, 17 (8.9%) arrived as multiples. Six different individuals transported multiple fishing probes, and multiple transport occurred during visits to both epigeal (*n* = 3 visits) and subterranean (*n* = 4) nests. The transport of multiple puncturing sticks was rare, documented on only one occasion. Chimpanzees were not observed to transport multiple beehive pounding clubs, other honey gathering tools, or leaf sponges.

As predicted, chimpanzees occasionally transported a puncturing stick plus a fishing probe to subterranean nests (*n* = 7 events). There were no instances of tool sets being transported to epigeal nests. These chimpanzees have been observed to use tool sets in honey gathering (Sanz and Morgan 2009), but pounding clubs and other tools used in honey gathering were not transported simultaneously.

### Variation in procurement strategy according to age and sex

Tool origin category proportions varied by age (Table 3). Mature chimpanzees (adults and subadults) transported a greater proportion of their tools to tool sites than did other age classes. Juveniles arrived only with fishing probes and (on one occasion) a beehive pounding club and overall exhibited more diverse tool procurement strategies. Infants were never observed arriving with any tool type.

**Fig. 3:**
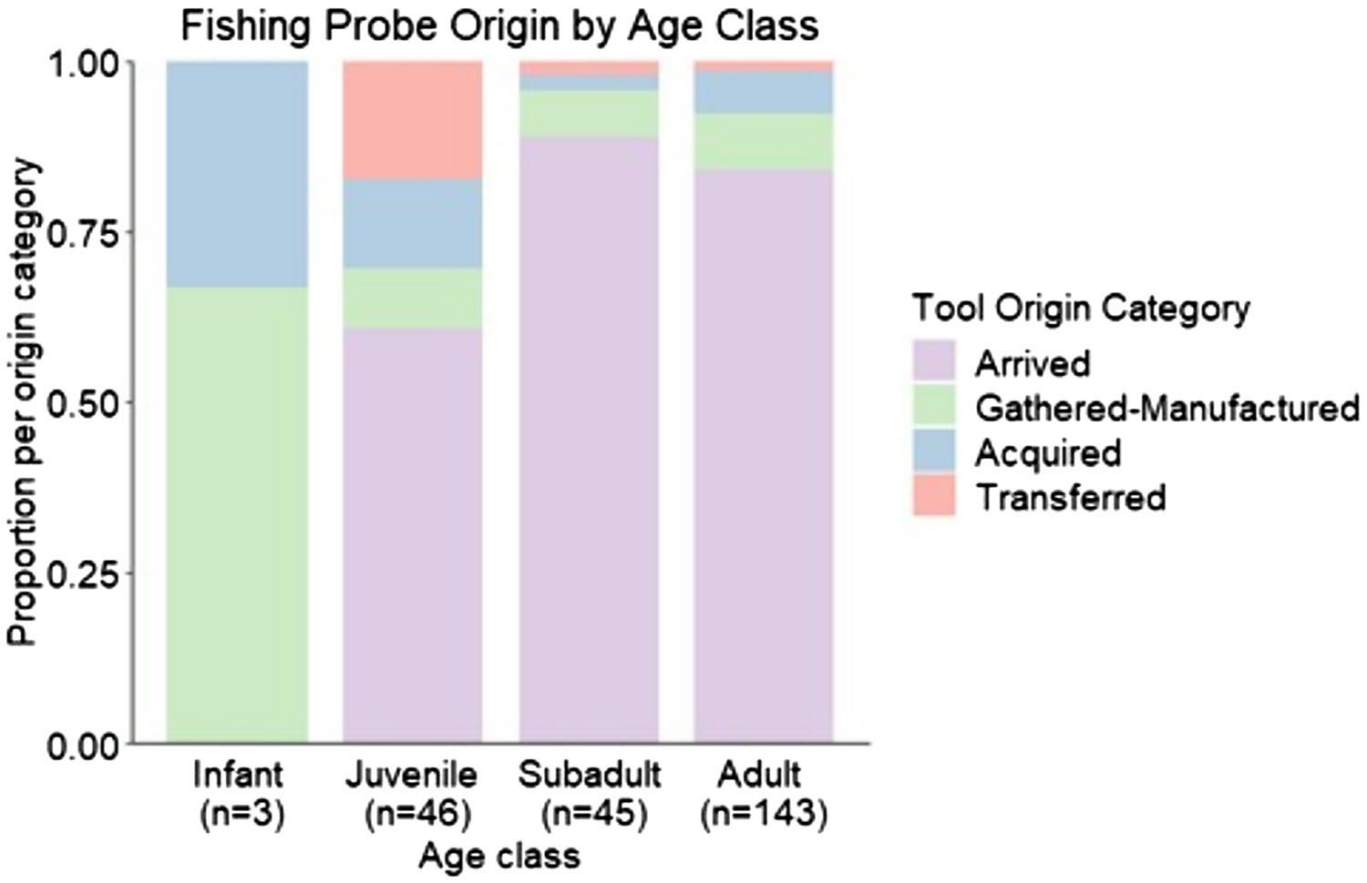
Fishing probe origin varied across age classes. While adults, subadults, and juveniles most often arrived with fishing probes, this strategy was significantly more likely for mature (adult and subadult) compared with immature (juvenile and infant) chimpanzees. Juveniles’ procurement strategy was more diverse, most notably with a greater proportion of these tools procured via transfer

For fishing probes specifically, there was a significant difference between mature and immature chimpanzees with respect to whether or not chimpanzees arrived with this tool type (χ^2^(1, *n* = 237) = 16.87, *p* < 0.0001). Arrivals comprised 85.1% of events (*n* = 160/188) for mature chimpanzees and 60.9% of events (*n* = 28/46) for juvenile chimpanzees. Mature and juvenile chimpanzees gathered and manufactured probes in similar proportions, but mature chimpanzees acquired these tools via transfer for only 1.6% of events (*n* = 3/188), compared with 17.4% (*n* = 8/46) for juveniles (Fig. 3). Only mature chimpanzees were documented transporting puncturing sticks to termite nests. Acquiring these tools at the nest after arrival was the most common strategy for both subadults and adults, with this category comprising 60.5% of events (*n* = 23/38). Most observations (88.9%) of perforating tool use involved adults, who either gathered and manufactured (*n* = 7/16 events, 43.8%) or picked up (*n* = 9/16 events, 56.3%) a perforating twig. There was one occurrence each of perforating twig manufacture by a juvenile and an older infant. Pounding of beehives was also primarily an adult activity, whereas use of other tools in honey gathering was most common among juveniles who gathered and manufactured these tools at the hive. We most often observed leaf sponging among subadults and adults, who gathered leaves that were within arm’s reach of the water basin to manufacture a sponge.

Tool origin category proportions were similar between mature males and females across tool types, though females arrived with the majority (*n* = 5/6 events) of beehive pounding clubs (Table 4). No significant sex differences were detected in the tendency to transport fishing probes to nests (χ^2^(1, *n* = 185) = 0.5, *p* = 0.48). Arriving with a fishing probe was the most common procurement strategy for both mature females (*n* = 93/107 events, 86.9%) and males (*n* = 64/78 events, 82.1%). Females and males also showed similar patterns for puncturing sticks and perforating twigs, with both sexes most often acquiring puncturing sticks on site and procuring perforating twigs by a combination of acquiring after arrival or manufacturing on site. For fishing probes, arrivals constituted *n* = 14/27 events (51.9%) for immature females and *n* = 4/8 events (50%) for immature males. All uses of puncturing (*n* = 3 events) and perforating (*n* = 2 events) tools among immature chimpanzees of known sex were by juvenile females. Both females and males exclusively gathered and manufactured leaf sponges on site.

**Table 4:** Number and proportion of events per tool origin category and sex for mature chimpanzees, by tool type

	Tool origin				
	Arrived	Gathered and manufactured	Acquired	Transferred	Total no. of tools
Fishing probe					
Females	93 (86.9%)	6 (5.6%)	7 (6.5%)	1 (0.9%)	107
Males	64 (82.1%)	9 (11.5%)	3 (3.8%)	2 (2.6%)	78
Puncturing stick					
Females	4 (22.2%)	1 (5.6%)	11 (61.1%)	2 (11.1%)	18
Males	4 (20.0%)	1 (5.0%)	12 (60.0%)	3 (15.0%)	20
Perforating twig					
Females	0	5 (50%)	5 (50%)	0	10
Males	0	2 (33.3%)	4 (66.7%)	0	6
Beehive pound					
Females	5 (83.3%)	1 (16.7%)	0	0	6
Males	0	1 (100%)	0	0	1
Other honey gathering tool					
Females	0	1 (100%)	0	0	1
Males	0	1 (100%)	0	0	1
Leaf sponge					
Females	0	6 (100%)	0	0	6
Males	0	6 (100%)	0	0	6
Total per category	170	40	42	8	260

As a group, adult females with dependent offspring arrived with termite-gathering tools (70.1% of events) at a similar proportion as did subadult and adult females without dependent offspring (75.0% of events). Similarity remained between the two groups when examining only fishing probes (85.7% versus 93%). However, a seemingly practical difference was that adult females with multiple immature offspring always arrived with their fishing probes (*n* = 10 events), as opposed to procuring them via other means.

### Availability of raw material for tool types that reflect selectivity

Botanical surveys at termite nests showed that the nearest stand of suitable herbs was > 20 m for 4/37 nests surveyed. Chimpanzees do not always use the closest available herbs, however. For example, for 21 of those 37 nests, the closest suitable raw materials did not show evidence of being used by chimpanzees, despite clear evidence of recent termite-gathering at the nest.

## Discussion

This research examined chimpanzee tool procurement behavior in the context of termite gathering at subterranean and epigeal termite nests, honey gathering, and leaf sponging. In both termite-gathering contexts, chimpanzees must solve a sequential challenge, but important differences between termite nest types and across tool types provide insights into whether and how chimpanzees plan their tool use. Tool procurement strategies varied depending on tool type and sometimes involved transport of multiples and tool sets, suggesting that chimpanzees flexibly planned for complex, sequential tool tasks. In contrast to the termite-gathering context, chimpanzees’ more flexible choice of materials for honey gathering and leaf sponging highlights links between raw material selectivity and procurement strategy, as chimpanzees did not often transport tools to these the sites where the tools will be used. We also documented that mature and immature chimpanzees exhibited differences in tool procurement strategies, indicating likely developmental correlates of tool use planning.

Consistent with what would be expected if tool use is planned, chimpanzees always arrived with tools specific to the termite nest type visited. For both subterranean and epigeal nests, a fishing probe is required to extract termites, and chimpanzees transported these to both termite nest types. Subterranean nests further necessitate the use of a puncturing stick before a fishing probe, and chimpanzees sometimes transported a puncturing stick in addition to a fishing probe to this termite nest type. It is noteworthy that chimpanzees did not arrive to subterranean nests transporting only a puncturing stick; this tool type on its own cannot facilitate successful termite gathering (Sanz et al. 2004). Nor did chimpanzees ever transport puncturing sticks to epigeal nests. These observations indicate that chimpanzees plan not only to gather termites, but to visit a particular type of termite nest (Byrne et al. 2013).

Chimpanzees also transported the different tool types in varying relative frequencies. Individuals most often gathered and manufactured fishing probes prior to arriving at the nest, and they did so significantly more often for fishing probes than for puncturing sticks. Given that chimpanzees source particular materials from the landscape to make fishing probes (Sanz and Morgan 2007), gathering the raw materials for these tools en route is likely more efficient than traveling to a termite nest and then leaving to search for the desired materials if they are not found growing in the immediate vicinity of the termite nest. Herb stems degrade quickly, so experienced termite fishers rarely pick up and reuse discarded fishing probes. The fragility of the brush tip may also prompt chimpanzees to at least occasionally transport multiple probes, in anticipation that one herb stem will prove insufficient for the duration of a termite fishing bout. While chimpanzees are also selective for materials used to make puncturing sticks, these woody tools are more durable than fishing probes, and they are conserved at subterranean nests for weeks or months (Sanz et al. 2004). The larger proportion of these tools procured via acquisition after arrival suggests that chimpanzees likely anticipate that that they can acquire and reuse puncturing sticks at subterranean nests. In contrast to both of these tool types, perforating twigs are only optionally used at termite nests, and various woody raw materials found in abundance are suitable for reopening the exit holes on the nest surface. Accordingly, chimpanzees never arrived with perforating twigs. Future research could explicitly quantify woody materials to further assess this interpretation, but the ease and rapidity with which chimpanzees source twigs suggest that source materials are consistently plentiful (e.g., Additional file 2: Video S2).

**Fig. 4:**
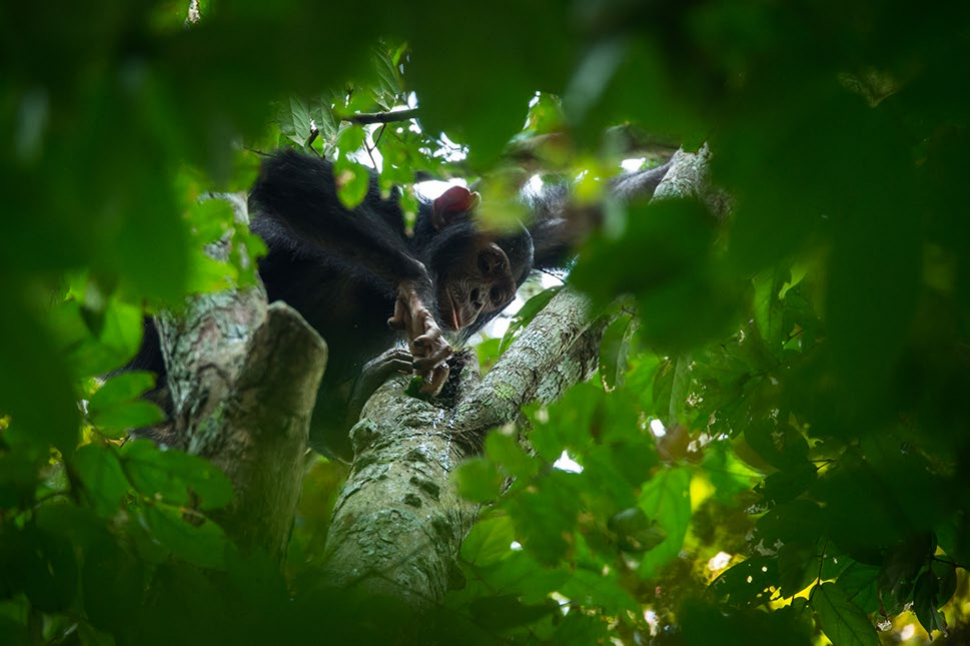
A juvenile male uses a leaf sponge to gather water from a tree basin. For this arboreal tool task, chimpanzees gather leaves from within arm’s reach of a water basin, without preference for specific plant species. Photo credit: Kyle de Nobrega, Goualougo Triangle Ape Project, Wildlife Conservation Society

**Fig. 5:**
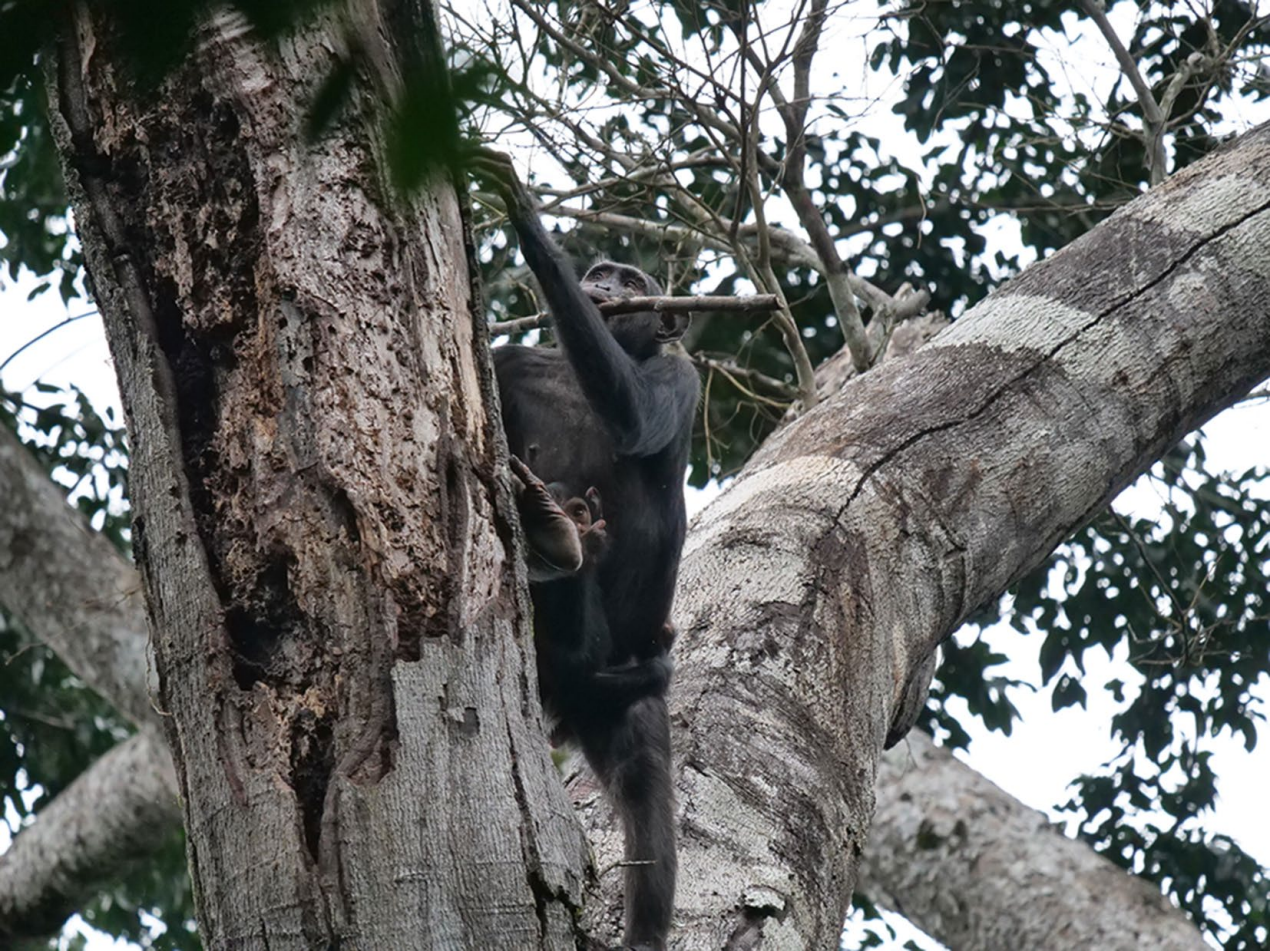
An adult female with a clinging infant transports a beehive pounding club to an arboreal beehive. In the present study, most observations of beehive pounding club transport were documented among mature females. Photo credit: Sean Brogan, Goualougo Triangle Ape Project, Wildlife Conservation Society

Suitable leaf-sponging materials also appear to be abundant, as chimpanzees gathered and manufactured these tools rather than transporting them in advance or acquiring them on site (Fig. 4). The opportunistic nature of leaf sponging, given that a water basin is likely more ephemeral than a termite nest, and the fragility of leaf sponges that precludes retaining them between visits, may also help account for differences across tasks. In the honey gathering context (Fig. 5), the balance of observations between the categories of arrival and gather and manufacture could reflect similar dynamics as well as the heterogeneous nature of beehives (Sanz and Morgan 2009), such that it is difficult for chimpanzees to anticipate precisely what form of tool will be best suited for gaining access to a hive. All of our observations of honey gathering and leaf sponging occurred in an arboreal environment, which likely also reduces opportunities for tools to be retained at tool sites and acquired after arrival. Increasing terrestriality during the Pliocene (Foley and Gamble 2009) is hypothesized to have facilitated acceleration of technological complexity among hominins, one reason for which could have been the enhanced opportunity to encounter others’ discarded tools (Meulman et al. 2012). Future research could compare how such constructed niches (Sanz et al. 2019) vary across terrestrial and arboreal settings.

In combination with other supporting evidence, the present data suggest that rather than routinely collecting and transporting tools in case an encounter with a termite nest occurs, or gathering tools only after encountering a termite nest, chimpanzees plan to visit a particular nest and thus procure the relevant tools efficiently, when they intend to use them. Planning for multiple tool use in this context could provide several advantages, including more efficient production of tools in adherence to a mental template of tool form. While transporting perishable tools is not as energetically costly as carrying nut-cracking hammers, for example, small improvements in efficiency of termite gathering may be nontrivial, especially given that termite-fishing sessions may be brief—on average, sessions last 6.05 ± 2.55 min (Sanz and Morgan 2013). In addition, in a social group, arriving with tools or with multiple tools could prevent an individual from being displaced from a site after leaving to gather materials, and mothers may avoid having to leave or retrieve infants when coming and going to gather raw material.

While in the present study we have classified our observations as minimally representing a case of planning for current needs (e.g., hunger), we emphasize that this planning may encompass anticipation of multiple future, sequential stages of a task. It is also possible that chimpanzees anticipate the need for tools at future times, even if they do not immediately intend to termite fish (i.e., the “spoon test,” Suddendorf and Corballis 1997; Tulving 2005). For example, chimpanzees at Goualougo have been observed to conserve termite-gathering tools during rest periods (Krause and Sanz 2019). In captivity, apes can save tools for use at later times (e.g., > 14 h wait, Mulcahy and Call 2006). Together, these findings suggest that chimpanzees can prepare for tool use over flexible time scales. Such flexibility is likely adaptive, given that the choice to use tools and the associated decisions regarding tool procurement may need to include dynamic evaluation of multiple ecological and social variables. For example, chimpanzees must efficiently locate and track availability of ripe fruit, which is widely and heterogeneously distributed on the landscape (Ghiglieri 1984); they must also monitor the location of conspecifics, including potentially hostile individuals from other communities (Wilson et al. 2014).

Continued research may help to illustrate aspects of this decision making, for example, by quantifying how far chimpanzees typically travel between other resource types (e.g., fruit or leaves) and termite nests or how chimpanzee visitation to specific locations in the forest relates to nearby resource availability (e.g., Janmaat et al. 2016) or to the ranging of conspecifics or other species, including other apes (e.g., Sanz et al. 2022). A full understanding of potential planning behavior requires consideration of these complementary abilities. In addition to spatial knowledge of one’s home range, understanding of tool functional properties likely also supports efficient identification of tool material and manufacture of tools. In captive experiments, all great ape species tested can differentiate functional and nonfunctional tools (Hermann et al. 2008), but continued examination of both interspecific (e.g., Visalberghi et al. 2015) and intraspecific (e.g. Boesch 2022) variation in the ability to select and transform raw materials is necessary to further clarify the flexibility underlying these behaviors, what specific physical properties are prioritized by tool makers, and how individuals acquire these skills (Musgrave and Sanz 2019).

An important methodological limitation of the present study is that due to the limited field of view of camera traps used to record termite-gathering activity, the complete behavioral sequence of gathering raw material, manufacturing a tool, and transporting the tool in this context is often not captured. As a result, it is not necessarily possible to infer the distance over which the tool was transported or to confirm whether the chimpanzee saw the termite nest prior to gathering raw materials. In some cases, however, camera traps do preserve such details, including instances of transport where the termite nest is not visible through the understory from where the chimpanzee comes into view (see Additional file 1: Video S1). Even when transport distance cannot be quantified, however, several lines of supporting evidence support the interpretation that tool manufacture and subsequent termite gathering are not simply prompted by seeing a termite nest. Observations of navigational behavior and use of “shortcuts” suggest the ability to exploit specific locations on the landscape (e.g., efficient deviations to arrive at ant-infested trees, Nishida and Hiraiwa 1982). In addition, chimpanzees at Goualougo have been directly observed procuring tool materials in several tool contexts and transporting tools between termite nests (Sanz et al. 2004; Sanz et al. 2007). Further, they routinely visit multiple termite nests in close proximity (but out of view from each other) on the same day (unpublished data). Such persistence is a hallmark of intentionality (e.g., Leavens et al. 2005), suggesting pursuit of an overarching goal. It is certainly possible, that individuals sometimes decide to termite gather at the moment that they encounter a suitable location. Altogether, however, multiple lines of evidence support the interpretation that chimpanzees routinely plan to gather termites and that at least sometimes, they prepare accordingly en route to the nest.

The limitations of the present study nonetheless suggest important avenues forward. Using direct or remote video observations in conjunction with measurements of retrieved tools could help to confirm the extent to which chimpanzees plan or adapt tools for the unique demands of particular nests (Sanz et al. 2014). Combining behavioral observations with insights from primate archaeology (Almeida-Warren et al. 2017; Carvalho and Almeida-Warren 2019; Haslam et al. 2017) will also provide expanded insights into tool procurement strategies and planning (Musgrave and Sanz 2021). For example, chimpanzees did occasionally arrive to subterranean nests with the complete tool set, comprising a puncturing stick and fishing probe.

Nonhuman apes are sometimes considered unable to gauge the certainty of their own memories (Suddendorf and Redshaw 2017). However, one potential explanation for occasional preparation of multiple tools is that chimpanzees account for their own uncertainty about the assemblage at a given location. Quantifying relevant variables such as time since an individual last visited, existing count of puncturing sticks present, availability of nearby suitable raw materials, and number of individuals in visiting party could help to further illuminate what contingencies guide decisions about transport of multiple tools and tool sets.

In addition to arriving with tool sets, chimpanzees sometimes arrived with multiple fishing probes. Mothers with multiple offspring always transported their tools in advance, and one adult female who arrived with her infant daughter transported three fishing probes to the termite nest. This female has been documented transferring fishing probes and puncturing sticks to her offspring on numerous occasions (Musgrave et al. 2016; Musgrave et al. 2020a, b). Chimpanzees with dependents may further anticipate transfers of these tools. Indeed, mature compared with immature individuals were more likely to transport tools in advance, while procuring tools via transfer occurred more often for immature compared with mature chimpanzees.

There were no general sex differences detected among adults in tendency to transport tools or multiple fishing probes, and experienced termite fishers of both sexes are likely aware of the potential fragility of fishing probes. Especially for lightweight termite-fishing tools, the modest sexual dimorphism in chimpanzee body size is unlikely to influence decisions regarding tool transport. Overall, we have observed that adult and subadult females are more engaged in honey gathering than males in the Goualougo Triangle (Sanz and Morgan 2009). Thus, it was not surprising that most of our observations of the transport of beehive pounding clubs were also by mature females. During daily follows, we also noticed that females revisited particular beehives over both shorter (days) and longer time scales (years), which could have influenced their decision to transport tools. Whether sexual dimorphism affects chimpanzees’ tool procurement strategies in this or other contexts, such as hammer selection during percussive tool use, is not yet known. In bearded capuchins (*Sapajus libidinosus)*, a highly sexually dimorphic species, median distances of hammer transport to crack encased foods appear similar between sexes. Thus, both males and females recognize and prefer heavier hammers despite the greater cost of transport for smaller-bodied individuals (Visalberghi et al. 2015). Rather than resulting from a clear single factor such as sexual dimorphism or cognitive differences, sex differences in chimpanzee tool use, including differences in procurement and planning, likely reflect complex interactions of multiple factors (e.g., physiological, socioecological, and developmental) depending on tool context, as well as individual differences.

The more limited evidence for planning behavior observed in immature chimpanzees could reflect cognitive limitations; in human children, robust future thinking is posited to develop only after the age of 4–5 years (Martin-Ordas 2020). Another possibility is that growing spatial independence influences tool procurement among younger chimpanzees. Infants, who travel on their mother, may be unlikely to dismount to independently gather raw material en route to a nest. That arrivals comprised 61% of procurement events for juvenile chimpanzees suggests that older dependents, however, are taking a more active role in preparing for tool use. Juvenile arrival events took several different forms, for example, juveniles locomoting independently yet arriving with a mature chimpanzee(s), usually their mothers, with both chimpanzees transporting fishing probes; juveniles, typically close to sub-adulthood, visiting a termite nest alone and transporting a fishing probe; and juveniles arriving with their mother, with only the juvenile transporting a fishing probe. These observations suggest that older dependents may take cues from mothers or other older individuals who are gathering raw materials but may also be influencing travel and tool use decisions. Future studies could further investigate mother–offspring communication and travel interactions in these contexts as they intersect with planning for tool use and other activities.

Ongoing research on how primates prepare for tool use will be essential for improving inferences regarding the scope and time depth of planning among human ancestors. Early hominins likely gathered tool materials and manufactured tools in advance of use (e.g., Braun et al. 2008), which may have enabled more efficient procurement and use of raw materials (Delagnes and Roche 2005). Planning could have conferred additional energy-saving benefits when tool makers sought specific raw materials. This selectivity is inferred when the prevalence of particular materials in archaeological assemblages exceeds their abundance on the landscape relative to other possible materials (Harmand 2009; Stout et al. 2005). Inferences based on this evidence, however, are complicated by time-averaging of the archaeological record (Luncz et al. 2016). Further study of flexible planning in living taxa closely related to humans is thus essential for reconstructing the evolutionary origins of these skills (Krause and Sanz 2019).

Flexibility is a key component of why future-oriented cognition is adaptive, with clear potential benefits in a range of contexts (e.g., Janmaat et al. 2014; van Schaik et al. 2013), including tool use and foraging. Increasing evidence suggests that planning capacities have likely convergently evolved across a range of distantly related taxa (Boeckle et al. 2020). Continued research will be essential for illuminating how planning skills develop over the lifespan and how planning behavior might vary between sexes or individuals or depend on specific task characteristics (e.g., energetic demands of tool transport, access to others tools in particular contexts, increased likelihood of success with tool sources from particular materials). Integrating behavioral, spatial, and archaeological data on tool use are important future steps for comparative study across taxa and to improve inferences about the decision-making processes and time depth of planning associated with the technologies of human ancestors.

## Supplementary Information

Below is the link to the electronic supplementary material.

Supplementary file 1: (MP4 170707 KB)

Supplementary file 2: (MP4 29549 KB)

## Data Availability

The data that support the findings of this study are available from the corresponding author upon reasonable request.
